# Knee Mechanics, Strength and Flexibility: Assessing Injury Risk in Female Adolescent Soccer Players

**DOI:** 10.3390/jfmk10010077

**Published:** 2025-02-25

**Authors:** Koulla Parpa, Marcos Michaelides

**Affiliations:** School of Sciences, University of Central Lancashire, Cyprus Campus, University Avenue 12-14, Pyla, 7080 Larnaka, Cyprus; mmichaelides@uclan.ac.uk

**Keywords:** Q angle, knee hyperextension, interlimb asymmetries, non-contact injuries, ACL, muscle strains

## Abstract

Background/Objectives: This study examined the link between the Q angle, knee hyperextension, flexibility, strength profiles and injury occurrence in female adolescent soccer players. Methods: Thirty adolescent female soccer players (age range: 15–17 years; age: 15.47 ± 0.73 years; weight: 55.91 ± 7.44 kg; height: 160.01 ± 5.58 cm) were recruited for the study. The tests were conducted before the pre-season preparation period, and the players were monitored from the beginning to the end of the season. This study included players who sustained non-contact injuries throughout the season as well as those who did not sustain any injuries for comparison purposes. Players underwent an anthropometric assessment (height, weight, body fat, Q angle, knee hyperextension) and completed a sit-and-reach test and an isokinetic assessment at 60°/s. Results: The results showed that 36.67% of players sustained a non-contact injury during the season. Based on the isokinetic assessment at 60°/s, significant differences were observed between the two groups in the torque production of the right and left knee extensors [t(28) = 2.32, *p* = 0.03, d = 0.81 (large effect)] and the right and left knee flexors [t(28) = 2.04, *p* = 0.05, d = 0.71 (medium effect)], with the injured group demonstrating significantly greater interlimb asymmetries in torque between the right and left knee extensors, as well as the right and left knee flexors. Also, the injured group demonstrated significantly higher knee hyperextension values for both the right [t(28) = 6.12, *p* < 0.05, d = 2.22 (large effect)] and left legs [t(28) = 5.72, *p* < 0.05, d = 2.15 (large effect)]. Conclusions: interlimb asymmetries and knee hyperextension may contribute to the occurrence of lower body non-contact injuries in adolescent female soccer players.

## 1. Introduction

Female soccer players are at an increased risk of injuries, particularly quadriceps strains and severe ligament injuries to the knee and ankle joints [1]. Research indicates that the total number of absence days is 21% higher in women compared to men, due to a 5.36 times greater incidence of severe knee and ankle ligament injuries [1]. In addition, quadriceps strains, anterior cruciate ligament ruptures and ankle syndesmosis have been shown to be more common in female soccer players than in their male counterparts [1]. Factors contributing to this increased risk include, but are not limited to, anatomical and structural differences between genders, hormonal effects and inadequate strength [2,3,4]. When assessing injury susceptibility in female athletes, factors such as knee hyperextension or genu recurvatum, the quadriceps angle (Q angle), knee valgus, hamstring flexibility, a high body mass index (BMI) and strength profiles might play an essential role in understanding anatomical and biomechanical predispositions to injuries [3,5,6].

Knee hyperextension or genu recurvatum, where the knee extends beyond its normal range, has been associated with an increased risk of anterior cruciate ligament injuries [7]. Concurrently, research indicates that knee hyperextension is linked to knee laxity, and both are recognized as intrinsic factors for non-contact anterior cruciate ligament (ACL) injuries [8]. During hyperextension, the tibial attachment of the ACL is prevented from moving forward by its extension-restraining fibers [9]. However, if the hyperextension force is large, these fibers can be overstressed, potentially leading to strain or even rupture. This could explain the increased risk of ACL injury, especially during unbalanced movements, sudden changes in position or uncontrolled landings. Furthermore, knee hyperextension has been associated with weaker hamstring strength and lower hamstring-to-quadriceps ratios in healthy male athletes [10]. At the same time, hamstring flexibility has been linked to high levels of knee laxity in injured players, suggesting that above-average hamstring flexibility may compromise the passive protective function of the hamstring muscles on the ACL [11]. Thus, hyperextension may result from both structural predispositions and muscular imbalances, necessitating a more detailed examination of strength profiles as well as other anatomical characteristics, such as the Q angle.

The Q angle, or quadriceps angle, is determined by drawing a line from the anterosuperior iliac spine to the center of the patella, and a second line from the center of the tibial tubercle to the center of the patella. The angle formed at the intersection of these two lines is regarded as the Q angle [12], with females generally having larger angles due to a wider pelvis structure. A larger Q angle (greater than 15–20 degrees) has been suggested to increase lateral patellar tracking, potentially leading to issues such as lateral patellar dislocation or increased lateral patellofemoral contact pressures [13], patellofemoral pain syndrome [14] or even knee injuries. Even though some studies suggest an association between pelvic width, the Q angle and ACL injuries [15], the results are contradictory in female soccer players [16]. A small-scale study indicated that among female soccer players, there were no significant differences in ACL tears based on the Q angle, pelvic width or intercondylar notch width [16].

In addition to the previously mentioned parameters, strength profiles may play a key role in understanding injury susceptibility [17]. Research indicates that muscular imbalances and strength asymmetries may contribute to knee injuries [18] or be present in patients recovering from ACL surgeries [19]. Therefore, it is suggested that these strength asymmetries or imbalances could further compromise knee stability, especially when combined with knee hyperextension and an increased Q angle. Research indicates that if the asymmetry between the knee extensors exceeds 10%, it increases the risk of musculoskeletal injuries as well as ligament and meniscus injuries [20]. Thus, this study aimed to determine whether Q angle magnitude, knee hyperextension, flexibility and strength asymmetries differ between injured and non-injured female adolescent soccer players. More specifically, it examined whether players who sustained non-contact lower limb injuries exhibited greater Q angles, increased knee hyperextension, reduced hamstring and lower back flexibility and greater lower body interlimb asymmetries compared to their non-injured counterparts. We hypothesized that players with a Q angle greater than 15 degrees and knee hyperextension exceeding 5 degrees would be more likely to sustain a lower limb injury. Furthermore, we hypothesized that players with reduced lower back and hamstring flexibility, as well as interlimb strength asymmetries in knee extensors and flexors (>10% difference in peak torque at 60°/s), would have a higher risk of non-contact lower limb injury during the season.

## 2. Materials and Methods

**Figure 1:**
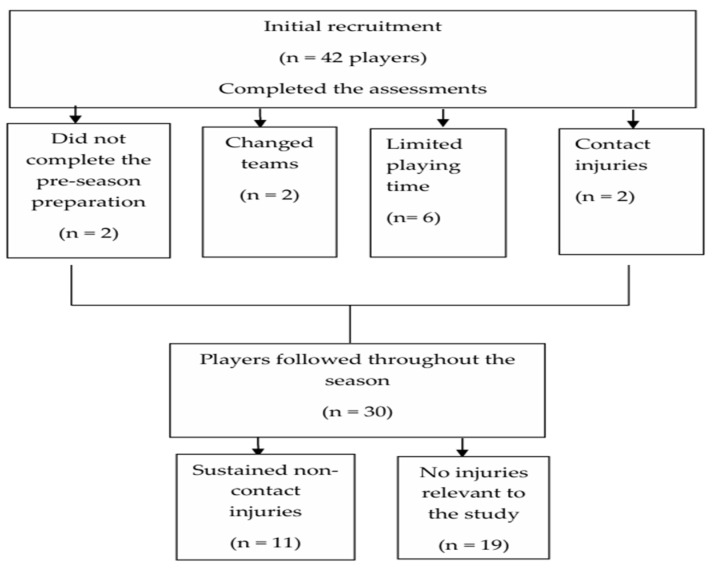
Participant inclusion and exclusion.

Thirty adolescent female soccer players (age range: 15–17 years; age: 15.47 ± 0.73 years; weight: 55.91 ± 7.44 kg; height: 160.01 ± 5.58 cm) from three teams participating in the U-18 Championship were recruited for this study. The initial testing was conducted on 42 players before the pre-season preparation period. However, only players who completed pre-season preparation and participated in all the friendly games were included in the analysis. Players with limited game time (non-starters) or irregular participation were also excluded (Figure 1). The remaining players (n = 30) were monitored from the beginning to the end of the season (approximately 18 games). Throughout the season, participants and the medical personnel of the participating teams were asked to report any injury that occurred during an official game or training that resulted in the player’s inability to continue participating. Also, they were asked to report the date of the injury, the body part involved and the mechanism. While the coaching team reported all injuries to the principal investigator, only lower body non-contact injuries directly relevant to the purpose of the study were included in the analysis. The injuries included in this study were those that were clinically diagnosed and led to an absence from training or competition for at least seven days. Furthermore, only non-contact lower body injuries that were classified as moderate (8–28 days of absence) and major (more than 28 days of absence) were included in the analysis. The classification of injuries was conducted according to the consensus statement on injury definitions and data collection procedures in studies of soccer injuries [21]. Therefore, this prospective cohort study included players who sustained non-contact injuries throughout the season as well as those who did not sustain any injuries for comparison purposes.

Regarding the performance assessments, the players completed a series of tests; however, only the parameters relevant to this study will be presented. All of the players had at least one week of training before the performance assessments, and they were advised to avoid heavy physical activities the day before testing, which was scheduled from 2:00 to 7:00 p.m. They all had previous experience with the testing procedures and participated in the study voluntarily after their parents or legal guardians signed an informed consent form. In addition to the anthropometric measurements (height, weight, body fat, Q angle, knee hyperextension), the players completed a sit-and-reach test and an isokinetic assessment at 60 degrees/s. Players who reported injuries within the six months before data collection were excluded from the study. This study was conducted in accordance with the Declaration of Helsinki and was approved by the National Committee of Bioethics (ΕΕΒΚ ΕΠ 2022.01.290, approved 29 January 2022).

### 2.1. Anthropometric, Body Composition Analysis, Q Angle and Knee Hyperextension Measurements

Stature was measured to the nearest 0.1 cm using a wall-mounted stadiometer (The Leicester Height Measure, Tanita, Tokyo, Japan). Body composition was assessed with a leg-to-leg bioelectrical impedance analyzer (BC 418 MA, Tanita, Tokyo, Japan). Before bioelectrical impedance testing, players were instructed to adhere to standard guidelines, including fasting for at least four hours, refraining from intense physical activity the day before the assessment, avoiding drinks with high caffeine content in the preceding twelve hours, and emptying their bladder before the test.

Following the body composition analysis, Q angle and knee hyperextension measurements were taken with the players in a supine position. For the Q angle measurements, the same experienced investigator provided instructions to the players and conducted all measurements twice. If there was a discrepancy between the measurements, a second experienced investigator (with more than 20 years of experience) verified the measurement. Players were instructed to lie supine with their knee extended (not hyperextended) while the investigator positioned the foot in a neutral position relative to supination and pronation, with the hip also in a neutral position relative to medial and lateral rotation. The investigator then drew a line from the anterior superior iliac spine to the midpoint of the patella and another from the midpoint of the patella to the tibial tubercle. The angle formed by the crossing of these two lines was measured as the Q angle with a Guymon digital goniometer (model 01129, Lafayette, IN, USA). 

Knee hyperextension measurements were also taken with a goniometer with the players in a supine position with both knees in extension. The players were instructed to actively tighten their quadriceps and fully straighten their knee. The center of the goniometer was positioned over the lateral epicondyle of the knee. The stationary arm aligned with the lateral mid-thigh, directed toward the greater trochanter, while the moving arm was aligned with the midline of the lower leg, directed toward the lateral malleolus. A full extension was recorded at 0°. Hyperextension beyond 0° was documented as a positive value, while an inability to fully extend the knee was noted as a limitation in knee extension. Measurements were obtained according to guidelines [22] by the same experienced investigator. Of note is that prior to the beginning of the study, two experienced investigators measured both the Q angles and knee range of motion of 12 female subjects to establish interrater reliability. The reliability values for the Q angle and knee hyperextension were 0.78 and 0.88, respectively.

### 2.2. Isokinetic Testing of Knee Flexors and Extensors

Isokinetic testing was conducted according to the methodology described by previous investigators [23,24]. Gravity correction was performed according to the manufacturer’s recommendations. The players were seated with their thigh positioned at an 85° angle to the trunk, while the dynamometer’s axis of rotation was aligned with the lateral epicondyle of the knee joint. The range of motion at the knee joint was 100°. Straps were used to secure the thigh, ankle and upper body. All participants had prior experience with the isokinetic device, as it is part of their regular testing.

Before testing, the players completed a 5 min warm-up on a mechanically braked cycle ergometer. Peak torque for knee flexors and extensors was measured at 60°/s using the HUMAC norm isokinetic device (CSMI, Stoughton, MA, USA). Once appropriately positioned on the isokinetic device, the players performed five repetitions for familiarization. The testing involved three maximal concentric flexion and extension repetitions at 60°/s, with the maximal torque from the three repetitions being used for further analysis. The same experienced examiner conducted both the set-up and testing.

### 2.3. Sit-and-Reach Test

A custom sit-and-reach box (32.4 cm high and 53.3 cm long) with a 26 cm heel line mark was used to assess the flexibility of the lower back and hamstring muscles according to methods described by previous investigators [25]. The players placed the soles of their feet (no shoes) against the box with knees in full extension. They were instructed to lean forward with one hand on top of the other and palms facing downward. Fast and jerky movements were not allowed while they were leaning forward. The players performed three attempts, and the best trial recorded to the nearest centimeter was entered for statistical analysis.

### 2.4. Statistical Analysis

Analysis was performed using SPSS, version 28.0, for Windows (SPSS Inc., Chicago, IL, USA). The normality assumption was assessed with the Shapiro–Wilk test (*p* > 0.05). All parameters are presented as the mean and standard deviations, as normality was confirmed. An independent sample *t*-test was conducted to compare the parameters of interest between the injured and non-injured players. Cohen’s d was utilized to determine the effect size. Effect sizes were interpreted as follows: small (0.2–0.4), medium (0.5–0.7) and large (0.8–1.4) [26]. Comparisons among the players who sustained different injuries were not possible due to the small number of players in each injury group. The level of significance was set at *p* < 0.05.

## 3. Results

**Figure 2:**
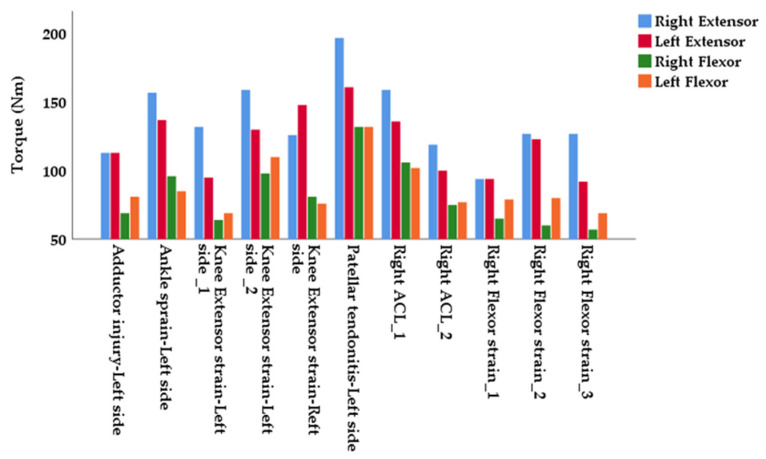
Extensor and flexor torque production and type of injury for the 11 injured players (one adductor injury, one ankle injury, three extensor strains, one case of patellar tendonitis, two ALC ruptures and three flexor strains).

The results showed that 36.67% of players (11 out of 30 players) sustained a non-contact injury during the season. Among these, three cases were moderate hamstring strains and three were moderate quadriceps strains. In addition, one adductor strain and one case of patellar tendonitis were classified as moderate injuries. One major ankle sprain (grade 3) and two ACL injuries (complete ruptures) were classified as major injuries (Figure 2).

The anthropometric and body composition parameters are presented in Table 1. Based on the isokinetic assessment at 60°/s, injured players generated significantly higher torque in the right extensors [t(28) = 2.04, *p* = 0.05, d = 0.74 (medium effect), Table 2]. Significant differences between the two groups were also indicated in the torque production between the right and left knee extensors [t(28) = 2.32, *p* = 0.03, d = 0.81 (large effect), Table 2] and the right and left knee flexors [t(28) = 2.04, *p* = 0.05, d = 0.71 (medium effect), Table 2], with the injured group demonstrating significantly greater interlimb asymmetries in torque between the right and left extensors, as well as the right and left flexors.

There were no significant differences in lower back and hamstring flexibility, as measured by the sit-and-reach test, between the injured and non-injured players. Similarly, no significant differences were observed in Q angle measurements between the two groups. However, the injured group demonstrated significantly higher knee hyperextension values for both the right [t(28) = 6.12, *p* < 0.05, d = 2.22 (large effect), Table 3] and left legs [t(28) = 5.72, *p* < 0.05, d = 2.15 (large effect), Table 3].

## 4. Discussion

This study examined the link between the Q angle, knee hyperextension, flexibility, strength profiles and injury occurrence in female adolescent soccer players. Based on the results, the injured group demonstrated significantly greater interlimb asymmetries in torque production between knee extensors, as well as knee flexors. Additionally, the injured group demonstrated significantly higher knee hyperextension values for both the right and left legs. In addition, a significant difference was observed in right extensor torque production, which disappeared when the results were adjusted for body weight.

In soccer, strength asymmetries have been associated with lower limb injuries, with researchers suggesting an increased risk of hamstring and quadriceps injuries in players exhibiting greater isokinetic peak torque asymmetries [27]. Specifically, it has been reported that the risk of musculoskeletal injuries in the lower extremities is 16 times higher when quadriceps torque asymmetry exceeds 10% and 12 times higher when hamstring peak torque asymmetry exceeds 10% [20]. Notably, the female players in our study demonstrated knee extensor and flexor asymmetries exceeding 10% (13.82 ± 9.71 and 11.91 ± 7.06, respectively), whereas the non-injured group demonstrated asymmetries below 10%. When one limb is weaker, it may not be able to stabilize the body effectively compared to the stronger limb, which may overload the weaker side and increase the likelihood of injury to the weaker side. At the same time, the stronger side may be subjected to excessive load in an attempt to compensate for the weaker limb, potentially leading to overuse injuries or strain of the stronger limb (Figure 2). Previous researchers have suggested that these asymmetries may result from the technical aspects of soccer games, which involve one-sided activities such as kicking and passing [28]. Additionally, longer training durations, particularly among professional players, have been associated with greater asymmetries [24]. In contrast, a Polish study investigating a large cohort of senior and junior soccer players demonstrated no significant differences in interlimb asymmetries between senior and junior players for any isokinetic strength measures. Concurrently, the investigators reported no intergroup differences for intra- or interlimb asymmetries when compared to normative values used to identify players at greater risk for knee injury [29]. A close look at the H/Q ratio in our study reveals a value of 59%, indicating a weaker hamstring muscle on the right side, which may explain some of the muscle strains or injuries (Figure 2). On the contrary, the remaining H/Q ratios were above 65%. Research on female athletes has reported that a reduced H/Q ratio, indicating weaker hamstrings relative to quadriceps, may be associated with an increased risk of ACL injuries [30]. That being said, a conventional H/Q ratio between 55 and 65% is considered to be within a safe range [20]. Considering that previous studies have not shown an association between the H/Q ratio and ACL or hamstring injuries, it is indicated that the H/Q ratio should not be used alone as a predictive tool for injury [31]. Also, while the previously mentioned studies and our work indicate an association between interlimb asymmetries, as measured by isokinetic testing, the results should be interpreted with caution as those asymmetries were assessed using slow isokinetic speeds which may not fully reflect the speeds generated during a soccer game. Also, there was considerable variability in the measurements. Therefore, it is recommended that practitioners and sports scientists assess the magnitude and direction of the individual interlimb asymmetry for a more accurate analysis. Nevertheless, the rationale for assessing interlimb asymmetries through isokinetic testing remains valid, as several studies, including ours, have demonstrated a link between these asymmetries and injury risk, although it must be acknowledged that the risk of injuries is multifactorial.

Regarding knee hyperextension, the injured group in our study demonstrated significantly higher knee hyperextension values for both the right and left legs. Although our study did not focus exclusively on ACL injuries, some of our results may still align with previous studies that suggest an increased risk of injuries, particularly non-contact ACL injuries, when the knee extends beyond the normal range [7,8]. More specifically, a study on young female athletes created a logistic regression, including knee hyperextension and side-to-side differences in anterior–posterior tibiofemoral translation, that was able to predict future ACL injury status [7]. The researchers suggested that a positive measure of knee hyperextension increased the likelihood of an ACL injury by five times [7]. In addition, a study that assessed knee joint hyperextension and joint laxity in patients who underwent ACL reconstruction reported that joint laxity was present in 42.6% of the patients, while knee hyperextension was observed in 78.7% of them [8]. Furthermore, the study reported that all patients with joint laxity also had knee hyperextension [8]. The authors concluded that ACL injury was more common in patients with joint laxity, particularly those with knee hyperextension [8]. However, given that our study examined a wide range of injuries, direct comparisons with studies specifically investigating ACL injuries should be made with caution. 

At the same time, research suggests that hamstring flexibility is associated with high levels of knee laxity in injured players, indicating that above-average hamstring flexibility may compromise the passive protective function of the hamstring muscles on the ACL [11]. However, our study did not support this finding, as there was no significant difference in lower back and hamstring flexibility between the injured and non-injured groups. Furthermore, excessive hyperextension may lead to the overstretching of the knee flexors and increased tensile stress, especially during eccentric contractions during the late swing phase of the running gait cycle, often leading to hamstring injuries during sprinting [32]. Also, research has demonstrated that increased knee hyperextension results in progressive damage to the translational and rotational knee soft tissue restraints [33]. This may have an impact not only on the anterior part of the knee joint but also on the posterior knee capsule, posterior cruciate ligament and hamstrings, which are responsible for preventing excessive knee extension [33]. If these structures become fatigued or overstretched, the knee becomes more susceptible to injuries such as ligament sprains, posterior ligament tears and meniscus damage.

## 5. Conclusions

Based on the results of this study, we conclude that interlimb asymmetries and knee hyperextension may contribute to the occurrence of lower body non-contact injuries in adolescent female soccer players. While it is debatable whether screening tests can reliably identify individuals at increased risk for sports injuries, these tests may still provide valuable insight. When used in combination, screening methods could enhance the ability to identify high-risk female soccer players by detecting specific biomechanical factors that predispose them to injury. Lastly, our results may help healthcare professionals implement targeted injury prevention strategies. Regarding interlimb asymmetries, coaching staff may implement target strength training programs using unilateral exercises to manage excessive asymmetries. Also, they may incorporate neuromuscular training in exercise routines to enhance coordination and reduce compensatory movements that may lead to injuries. Considering our small sample size that does not allow for accurate conclusions regarding injury directionality, it is recommended to monitor asymmetries over time and assess them in combination with other risk factors such as training load, movement mechanics and previous injury history. Also, rather than attempting to correct all asymmetries, it is recommended to address those that are excessive, get worse with time, and are linked to specific performance limitations or injury patterns. For knee hyperextension, coaching personnel may include additional strength training focused on the hamstring, glutes and calf muscles to improve joint stability. Last but not least, medical personnel could recommend prophylactic bracing depending on the severity of knee hyperextension.

### Limitations

This study comes with several limitations. First, the small sample size did not allow for comparison among players with different injuries. Also, due to the small sample size and short duration of the study, we did not have adequate statistical power to calculate injury rates. Furthermore, hormonal and dietary factors, which play an essential role in injury risk, were not considered. Also, the menstrual cycle was not recorded at the time of the injury, which could be a potential factor in understanding injury risk. The isokinetic measures are also presented without reference to the dominant or non-dominant legs, which might have influenced the results. Previous injuries were not reported, and the variation in pre-season programs and in-season training regimens (with varying training loads) further limits this study. Last but not least, considering that strength levels may fluctuate between the time of the isokinetic assessment and the occurrence of injury, the results of isokinetic testing should be interpreted with caution. Future studies with a longer duration and a larger sample size are recommended to determine whether asymmetry directionality correlates with injury occurrence.

## Figures and Tables

**Table 1:** Anthropometric and body composition characteristics (mean ± SD).

Parameter	Entire Group	Injured Group	Non-Injured Group
	(n = 30)	(n = 11)	(n = 19)
Age (years)	15.47 ± 0.73	15.45 ± 0.82	15.47 ± 0.70
Weight (kg)	55.91 ± 7.44	58.04 ± 8.06	54.68 ± 6.98
Height (cm)	160.01 ± 5.58	160.60 ± 6.27	159.67 ± 5.29
BMI (kg m^−2^)	21.85 ± 2.45	22.51 ± 2.53	21.47 ± 2.39
Body Fat %	24.34 ± 5.36	24.63 ± 6.28	24.17 ± 4.93

Note: BMI—body mass index.

**Table 2:** Lower body strength in injured and non-injured players (mean ± SD).

Parameter	Injured Group	Non-Injured Group	Cohen’s d
	(n = 11)	(n = 19)	
Right extensors 60°/s (nm)	137.27 ± 28.39	119.00 ± 20.53 *	0.74
Right extensors 60°/s normalized (nm/kg)	2.36 ± 0.34	2.18 ± 0.31	
Right flexors 60°/s (nm)	82.09 ± 23.39	77.00 ± 15.90	
Right flexors 60°/s normalized (nm/kg)	1.40 ± 0.26	1.41 ± 0.23	
Left extensors 60°/s (nm)	120.82 ± 23.76	113.42 ± 18.72	
Left extensors 60°/s normalized (nm/kg)	2.07 ± 0.24	2.08 ± 0.30	
Left flexors 60°/s (nm)	87.27 ± 19.49	80.63 ± 14.90	
Left flexors 60°/s normalized (nm/kg)	1.50 ± 0.21	1.48 ± 0.21	
H/Q ratio right side (%)	59.55 ± 8.77	65.32 ± 10.86	
H/Q ratio left side (%)	72.73 ± 10.08	73.47 ± 12.36	
Right and left extensor asymmetry (%)	13.82 ± 9.71	7.37 ± 5.59 *	0.81
Right and left flexor asymmetry (%)	11.91 ± 7.06	7.79 ± 4.08 *	0.71

Note: * *p* < 0.05; H/Q ratio—hamstring-to-quadriceps ratio.

**Table 3:** Flexibility, Q angle and knee hypertension in injured and non-injured players (mean ± SD).

Parameter	Injured Group	Non-Injured Group	Cohen’s d
	(n = 11)	(n = 19)	
Flexibility (cm)	30.38 ± 9.30	32.05 ± 7.50	
Q angle right (°)	9.73 ± 1.42	10.00 ± 1.25	
Q angle left (°)	10.09 ± 1.30	9.84 ± 1.30	
Knee hyperextension right (°)	9.91 ± 2.17	5.68 ± 1.60 *	2.22
Knee hyperextension left (°)	9.64 ± 2.01	5.47 ± 1.87 *	2.15

Note: * *p* < 0.05; Q angle—quadriceps angle.

## Data Availability

Data can be made available upon request to the corresponding author.
